# Outcomes and instruments used in social prescribing: a modified umbrella review

**DOI:** 10.24095/hpcdp.44.6.02

**Published:** 2024-06

**Authors:** Maureen C. Ashe, Isis Kelly dos Santos, Hadil Alfares, Anna M. Chudyk, Elham Esfandiari

**Affiliations:** 1 Department of Family Practice, The University of British Columbia, Vancouver, British Columbia, Canada; 2 Edwin S.H. Leong Centre for Healthy Aging, The University of British Columbia, Vancouver, British Columbia, Canada; 3 Department of— Physical Education, Federal University of Rio Grande do Norte, Natal, Brazil; 4 College of Pharmacy, University of Manitoba, Winnipeg, Manitoba, Canada

**Keywords:** determinants of health, healthy aging, outcomes research, social prescribing

## Abstract

**Introduction:**

Previous social prescribing work highlights a range in the types and number of outcomes used in published studies. We aimed to describe social prescribing outcome core areas and instruments to build capacity for future research and program evaluation.

**Methods:**

This was a modified umbrella review following standard guidelines. We registered the study and searched multiple databases (all languages and years); inclusion criteria were peer-reviewed publications containing outcomes for self-described social prescribing for adults aged 18 years and older. The last search date was 9 July 2023. From the included systematic reviews, we identified primary studies using the same inclusion criteria. For primary studies, we sorted extracted outcomes and instruments into six core areas using a published taxonomy. We located information on instruments’ description and measurement properties and conducted two rating rounds for (1) the quality of systematic reviews and (2) reporting of instruments in primary studies. We conducted a narrative synthesis of reviews, primary studies and outcomes (PROSPERO 2023 CRD42023434061).

**Results:**

We identified 10 systematic reviews and 33 primary studies for inclusion in our review. Outcomes covered most core taxonomy areas, with an emphasis on psychosocial factors (e.g. well-being) and less emphasis on cognition, physical activity, and caregivers and volunteers. We noted few studies provided detailed information on demographic data of participants or measurement properties of instruments.

**Conclusion:**

This synthesis provides an overview and identifies knowledge gaps for outcomes and instruments used in social prescribing interventions. This work forms the basis of our next step of identifying social prescribing–related outcomes that matter most across interested parties, such as individuals providers and decision makers.

**Highlights**

- We synthesized and categorized outcomes and instruments identified in 10 reviews and 33 primary studies for social prescribing.
- We highlight a range in the types and number of outcomes used in published studies.
- Many studies focussed on wellbeing and mental health outcomes.
- We noted less emphasis on use of outcomes for cognition, physical activity, and caregivers and volunteers.
- The field would benefit from comprehensive reporting of participants’ demographic information.

## Introduction

Social prescribing is a health and social model of care with origins in the United Kingdom (UK) and is quickly spreading to many other locations around the globe, including Canada. It has been well described elsewhere,^1^,^2^ but a key feature of social prescribing is the addressing of people’s unmet nonmedical social needs by connecting them to resources within the community. The most current comprehensive pathway for social prescribing^2^ involves health and social providers, or community organizations working with people to identify social needs and making a nonmedical referral to a community navigator, sometimes called a “community link worker.” Together, within a strengths-based approach, the person and link worker identify “what matters most” and the link worker will connect, or even in some cases accompany, people to community assets such as a service, greenspace or network (depending on needs).^2^,^3^

Social prescribing is informed by other evidence-based work, for example, community referrals, navigator systems,^4^ and the benefits associated with engaging in activities such as physical activity^5^ and arts-6 or museum-based programs.^7^ However, we^8^,^9^ and others^10^ have noted definitive evidence is lacking on the effectiveness of social prescribing. One challenge to synthesizing evidence may be due to the previous lack of an accepted definition of social prescribing; however, a new definition is available, co-created through an international modified-Delphi approach.^3^ In Canada, social prescribing is in the early stages of development, but is well supported by clinicians’ use of community referrals in practice and the large community-based sector of nonprofit, volunteer and other organizations that support people. The “new” definition and pathways of social prescribing are complemented by the integration of the health and social sectors and the strengths-based and person-centred approaches.^2^

Developing an evidence base for a complex intervention such as social prescribing requires considering many factors. One area we identified from our previous reviews,^8^,^9^ which may be useful in advancing the science and practice of social prescribing, is related to the outcomes measured within research and program evaluations. Inconsistencies in using and reporting outcomes within trials and programs present challenges when determining the effectiveness of an intervention (such as social prescribing), ensuring person-centred, meaningful and important outcomes are included, and, later, when combining data for evidence syntheses. 

Two previous studies used mapping review methods to identify and categorize outcomes in social prescribing.^11^,^12^ While both reviews provide important knowledge and perspectives, we proposed to go further and identify outcomes used in social prescribing studies and compare them against a recently published taxonomy of outcomes for health and social interventions from Dodd and colleagues.^13^ Reviewing currently used outcomes provides the opportunity to determine if any core areas or domains are missing from data collection and to describe current reporting practices.

Therefore, in this modified umbrella review, we aimed to describe outcomes, domains and instruments used in previous social prescribing studies for adults and older adults. The United Kingdom (UK) National Health Service (NHS) developed a Social Prescribing Common Outcomes Framework,^14^ but to our knowledge there is not a core outcome set for social prescribing research. We proposed to address this knowledge gap,^15^ and describe outcomes used in previous social prescribing research in advance of developing a core set.^16^ Taken together, we approached this work to provide practical guidance for choosing outcome measures, with the overall aim of contributing to the science that underpins social prescribing.

## Methods

We conducted a modified umbrella review following the guidelines outlined by the Preferred Reporting Items for Systematic Reviews and Meta-Analyses (PRISMA) statement^17^ and the Joanna Briggs Institute (JBI) standards for conducting an umbrella review, or review of reviews.^18^ We modified the review process by screening (via two authors, independently) each primary study within included reviews to confirm it met our inclusion criteria. We made the changes for two main reasons: (1) there was a wide age range and grey literature within the identified systematic reviews, and some of the primary studies did not meet our inclusion criteria; and (2) there was an overlap of primary studies across included reviews.

We registered the protocol with the international Prospective Register of Systematic Reviews (PROSPERO; 2023 CRD42023434061)^19^ before conducting searches with our final strategy. Our two exploratory research questions were: (1) What outcomes have been used in social prescribing research for community-dwelling adults aged 18 years and older? and (2)How do the identified instruments map onto the taxonomy of outcome core areas and domains?

***Eligibility criteria***

We included peer-reviewed systematic reviews and primary studies from all languages and all years that synthesized quantitative data for self-described social prescribing interventions for adults aged 18 years and older, and across the continuum of settings, such as hospitals primary care, community settings, etc. We included programs that have been described as “social prescribing,” such as arts-based or museum-based programs (known as “arts-on-prescription” or “museum-on-prescription”). We included evidence for adults and older adults for this synthesis, as this was our focus for developing a core set of outcomes. Further, younger and older populations have distinct needs and services, and therefore we did not include outcomes from younger age groups in this synthesis. 

We excluded publications that did not provide any outcome measures. We made the decision to include only peer-reviewed literature, because we wanted to compare reporting in the primary studies, which may be more likely to follow research reporting guidelines. 

The following were our criteria based on the population, intervention, comparator and outcome (PICO) framework. Population: we focussed on peer-reviewed evidence that included adults aged 18 years and older receiving social prescribing. Intervention: we included reviews and primary studies that self-identified as social prescribing. Comparator: studies included in the reviews could have any or no comparator. Outcomes: as our aim was to identify all possible outcomes, we did not place any limits on this component. 

***Information sources and search strategy***

We searched the databases listed below; the date of our last search was 9 July 2023. One author (MCA) ran all of the searches and uploaded identified citations into Covidence systematic review software (Veritas Health Innovation, Melbourne, AU). We also conducted a forward and backward (reference list) search for peer-reviewed publications from all included reviews based on citations downloaded from Web of Science or Google Scholar. The databases, along with keywords, were:

- Ovid MEDLINE and Epub Ahead of Print, In-Process, In-Data-Review & Other Non-Indexed Citations, Daily and Versions and Embase. Keywords: ("social prescribing" or "social prescription"). ab,ti. AND "systematic review".ab,ti.
- EBM Reviews - Cochrane Database of Systematic Reviews. Keywords: "social prescribing" OR "social prescription" AND "systematic review"
- EBSCO (APA PsycArticles, APA PsycInfo, CINAHL Complete, Social Work Abstracts, SPORTDiscus) social prescribing or social prescription (title) AND systematic review
- Epistemonikos "social prescribing" OR "social prescription" AND "systematic review" title and abstract
- Google Scholar "social prescribing" OR "social prescription" AND "systematic review" title

***Selection process***

All systematic reviews identified through search strategies were independently screened at Level 1 (titles and abstracts) and Level 2 (full text) by two authors (IKS, MCA) based on the inclusion criteria described above. We reviewed the primary studies from each included review and categorized them as peer-reviewed or unpublished studies/evaluations. We then reviewed the primary studies (e.g. a separate round of Level 1 and 2 screening) to decide if they met our inclusion criteria.

***Data collection process***

We extracted the following information for each review: author, publication year, systematic review question(s) and aims, population, setting, demographic information, summary of findings, and outcome measures or instruments. We also compared across reviews to look for overlap of primary studies to better understand data contributing to findings. For each included peer-reviewed primary study, we extracted the following information: author, publication year, population, setting, social prescribing intervention, and descriptive and outcome data collected. For this phase, one author (IKS or MCA) extracted information from studies in Covidence and Excel, and two other authors (EE and HA) reviewed and confirmed extracted findings. In the case of discrepancies between reviewers, a third review author (AC) made the final decision.

***Sorting process***

For each primary study, we extracted data on quantitative outcomes and sorted them based on a published taxonomy;^13^ we chose this taxonomy because it was developed to use in determining core outcome sets. The original taxonomy has five core areas: death, physiological and clinical, life impact, resource use, and adverse events. Within the core areas there are 38 categories or domains. Two authors (IKS, MCA) independently sorted outcomes into core areas and domains following the guidelines provided by the taxonomy,^13^ with two modifications: (1) we changed the domain “psychiatric outcomes” to “mental health” in the physiological/clinical core area; and (2) we moved the domain “delivery of care” to its own core area. 

One author (MCA) created the first table of sorted outcomes from the previous step, and then all other authors (IKS, EE, HA, AC) reviewed the table. We also reviewed and compared the extracted outcomes with the NHS Social Prescribing Common Outcomes Framework,^14^ which has four main areas: impact on the person (control and well-being, physical activity, management of daily life activities, connection); impact on community groups (confidence, impact of taking referrals, impact of social prescribing, changes in number of volunteers, capacity of the volunteer sector, and support needed); impact on the health and social care system (provider visits, medications, “morale of staff in general practice and other referral agencies”^14^^,p.30^); and other data collection (referrals, “equality monitoring,”^14^^,p.31^ contacts with link workers, satisfaction).^14^ Finally, for extracted instruments, we located information on measurement properties for a similar population (community-dwelling adults), when possible.

***Assessment of systematic reviews and primary studies***

We used the JBI critical appraisal tool^18^ to analyze systematic reviews included in this synthesis. For each primary study, we compared the outcome reporting against one criterion proposed by the Consolidated Standards of Reporting Trials (CONSORT) 2020 Extension for Outcomes:^20^ “Item 6a.8. Provide a description of the study instruments used to assess the outcome (e.g. questionnaires, laboratory tests) along with reliability, validity and responsiveness in a population similar to the study sample.”^20^^,p.2254^ We reviewed each study to locate the term within the publication; this could include either stating an instrument was valid, reliable and/or responsive, or providing a reference or measurement statistic. We also compared extracted demographic information for each primary study with the criteria proposed by PROGRESS-Plus^21^: age, gender/sex, ethnicity/culture/language/race, education, occupation, place of residence, religion, social capital and socioeconomic status.

***Synthesis methods***

We conducted a narrative synthesis, including compiling and sorting a list of outcomes and instruments contained within reviews and primary studies.

***Potential review biases***

We considered bias throughout our review process. A priori, we tried to address potential bias by following standard procedures and registering and updating our protocol. We planned for conflicts of interest, such as if an author on this synthesis were also to be an author on an included review or primary study. In this case, another author, not in conflict of interest, would provide a rating of the review. 

Team membership consisted of trainees and researchers, and two of the co-authors had clinical training. Our team also spanned the age range from young adult to older adult; team members had experience with multiple research methods, and authors with experience in systematic reviews mentored less experienced team members. However, none of the authors had direct, lived experience with social prescribing as defined in this work. 

We acknowledge that we only included peer-reviewed studies, and that this may create a publication bias, but this was intentional in order to identify and evaluate the reporting for outcome measures for studies that usually follow standard reporting guidelines (e.g. CONSORT 2020, or similar statements based on different study designs). However, by not including unpublished literature, we may have missed some outcomes, in particular as they may relate to implementation of a program (e.g. via a process evaluation).

## Results

***Study selection***

After two rounds of Level 1 and 2 screening, we included 10 systematic reviews (Figure 1A) and 33 primary studies (Figure 1B).

**Figure 1a:**
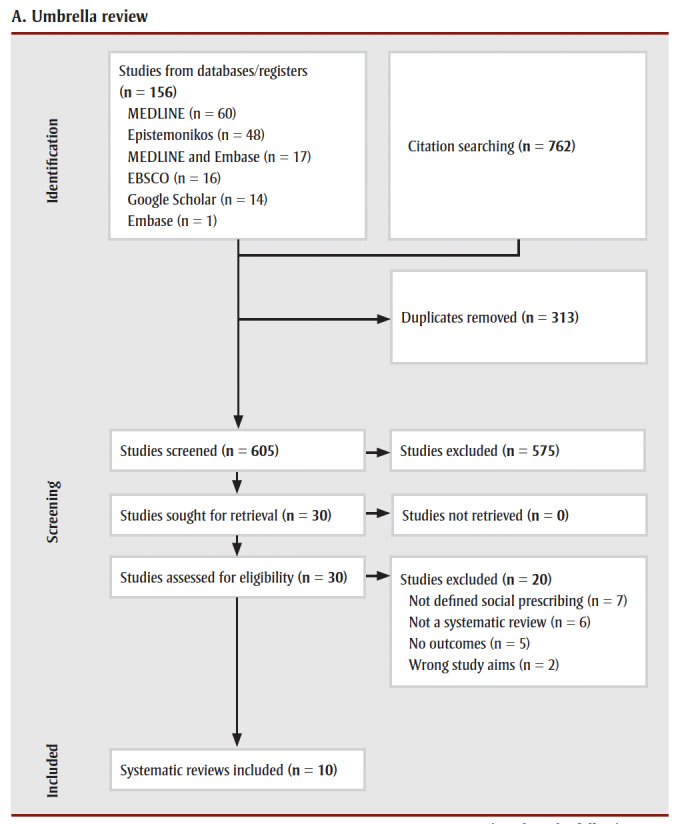
PRISMA flow diagram for umbrella review (A) and individual studies (B)

**Figure 1b:**
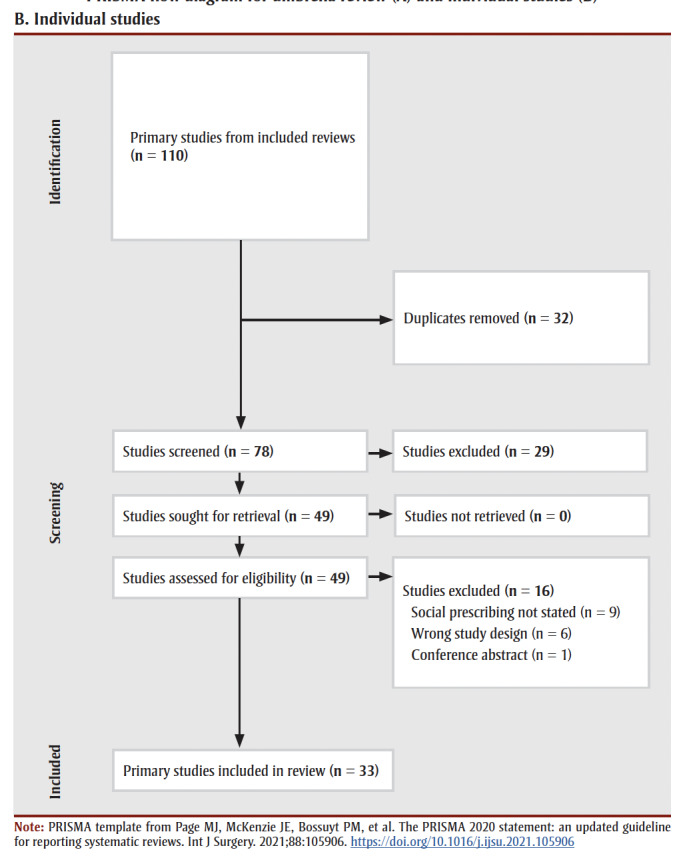
PRISMA flow diagram for umbrella review (A) and individual studies (B)

***Systematic review characteristics (n = 10)***

There were six systematic reviews with authors located in the UK,^10^,^22^-^26^ and one study each from Canada,^8^ Germany,^27^ Ireland^28^ and Portugal.^29^ Table 1 provides a summary of the systematic reviews, including the study aims and the number of included primary studies. Overall, participants’ descriptive information was missing from several systematic reviews;^10^,^25^,^26^ however, this may be because the information was missing from some of the primary studies. 

**Table 1:** Summary of information for the 10 included systematic reviews

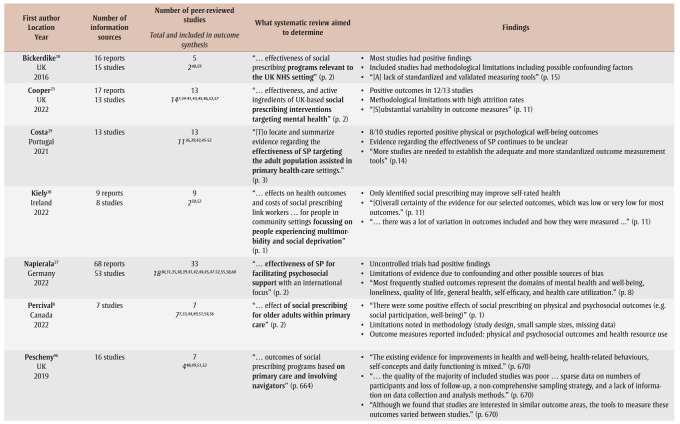 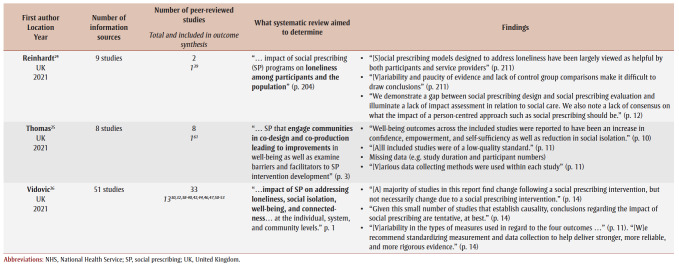

Almost all of the reviews aimed to look at the general effect or impact of social prescribing, with some reviews reporting more specific criteria such as social prescribing from one location (UK),^10^,^23^ or focussed on loneliness,^24^,^26^ mental health,^23^ primary care^8^,^22^,^29^ or older adults.^8^ One review focussed on social prescribing interventions that used a co-design or co-production approach.^25^ The review by Vidovic and colleagues^26^ provides an overview of outcomes used in social prescribing for four key measures: loneliness, social isolation, well-being and connectedness. Systematic reviews consistently noted positive outcomes but with limited evidence due to small sample sizes and methodological challenges with primary studies (Table 1). Most systematic reviews commented on the variability in the outcome measures.^10^,^22^,^23^,^25^,^26^,^28^,^29^

***Primary study characteristics (n=33)***

Table 2 provides an overview of the 33primary studies. Overall, 29 (88%) studies were conducted in the UK; three studies were from Australia,^30^-^32^ and one was from South Korea.^33^ Studies were published between 2008 and 2022, with 19 (58%) studies published from 2019 onwards, but before the publication of the social prescribing definition by Muhl and colleagues in 2023.^3^ Four studies^34^-^37^ were based on arts-on-prescription, “… part of mainstream social prescribing provision in primary health care.”^37^^,p.1^ Two studies were based on museums-on-prescription.^7^,^38^ Two studies^39^,^40^ were based on the social cure approach, which “suggests that social identities can enhance health/well-being through psychological resource provision.”^40^^,p.387^ We noted overlap for studies included in the 10 systematic reviews. Over half of the primary studies 19 (58%) appeared in more than one review, with a range in the number of times they were included: two;^7^,^30^,^35^,^36^,^40^-^43^ three;^7^,^44^-^47^ four;^48^-^50^ five;^39^,^51^ and six.^52^

**Table 2:** Summary of information for the 33 primary studies

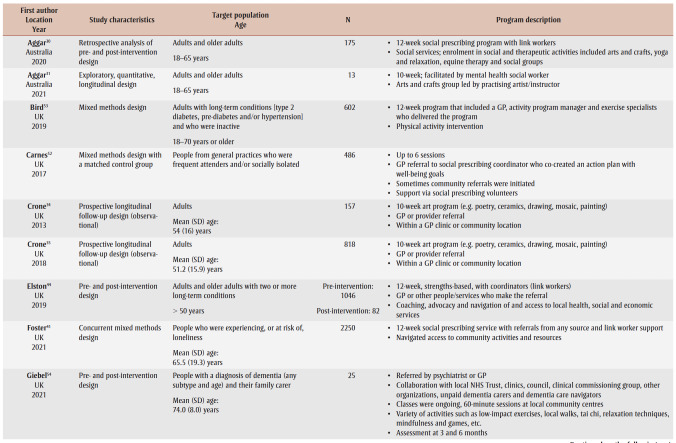 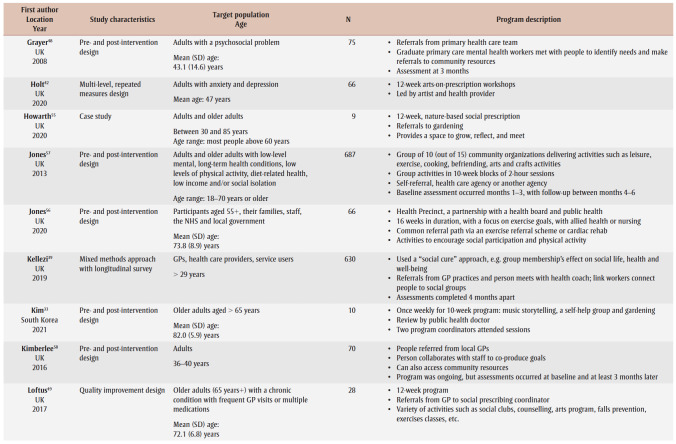 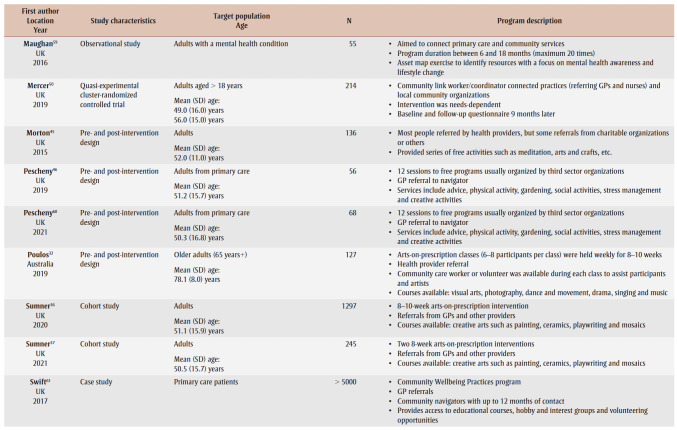 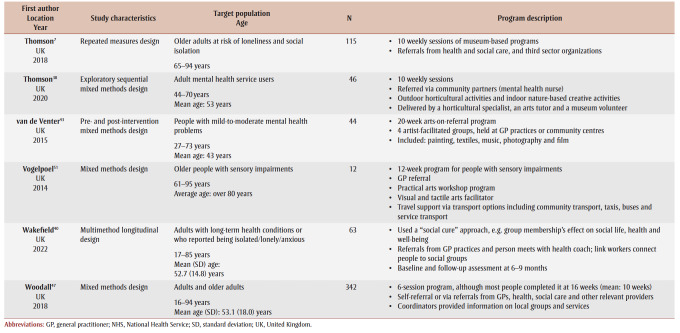

***Assessment of systematic review methods and instrument reporting***

Most systematic reviews were in agreement with the JBI critical appraisal criteria,^18^ with all but two reviews^24^,^26^ scoring eight or higher (out of 11 possible points). The question (from the JBI tool) rated with the most “no” or “unclear” responses was related to reporting the research question based on PICO format. Of primary studies, most publications did not provide detailed information on instruments’ basic measurement properties. Validity was most often mentioned or referenced,^7^,^30^-^34^,^36^,^37^,^41^-^44^,^47^,^48^,^50^,^51^,^53^-^59^ followed by reliability^33^,^36^,^38^,^42^,^43^,^48^,^53^,^54^,^57^ and responsiveness.^37^,^42^,^45^,^60^ Few studies provided specific information, such as a measurement statistic (e.g. Cronbach alpha or kappa).

***Results of synthesis***

**Categorization of outcomes**

**Figure 2:**
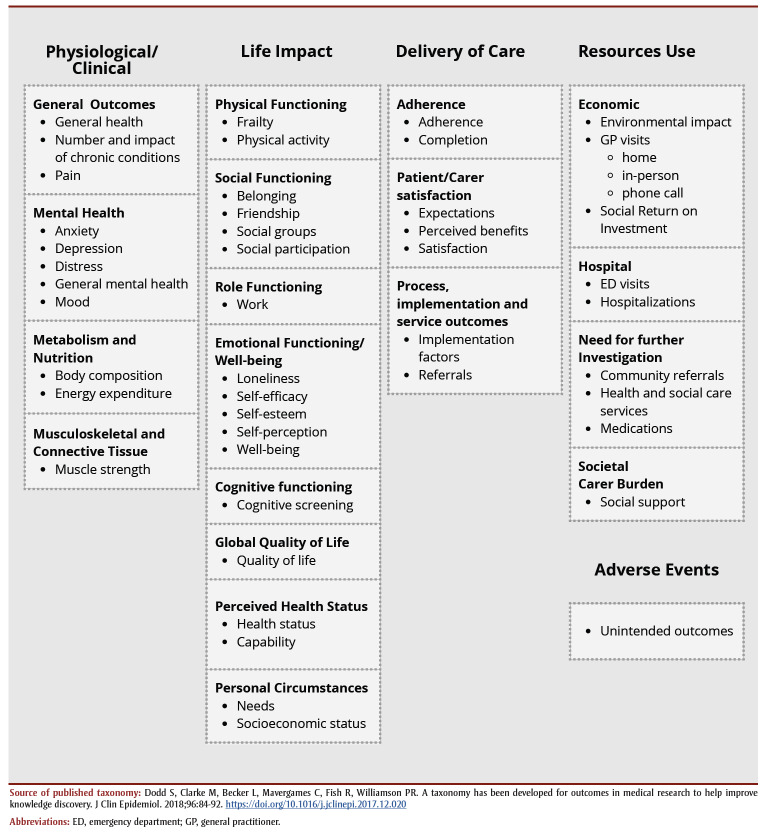
Overview of outcomes and information from primary studies based on the published taxonomy

Many extracted instruments were patient-reported outcomes measures (PROM) focussed on well-being, with variability in the number and types of outcomes used; there were some patient-reported experience measures (PREM; e.g. satisfaction). Figure 2 and Table 3 provide an overview of outcomes and information from primary studies. Overall, almost all taxonomy core areas were represented, except death, but most domains contained instruments from only a few studies (e.g. physical functioning such as physical activity), with some exceptions. 

**Table 1:** Outcomes extracted from primary studies, categorized using the published taxonomy, by core area and domain, showing instrument and citing primary study

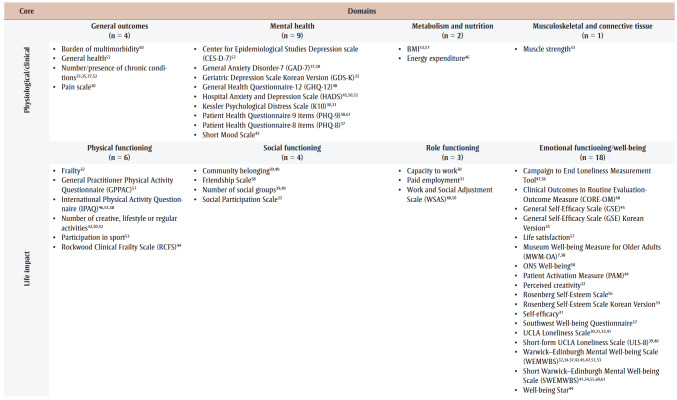 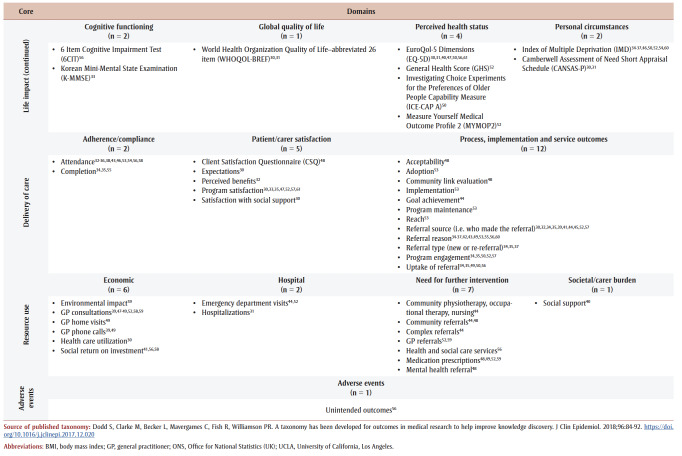

Specifically, over half of primary studies used at least one of six different well-being instruments, although most studies used one of two outcome instruments: Warwick-Edinburgh Mental Well-being Scale (WEMWBS)^32^,^34^-^37^,^42^-^45^,^47^,^51^,^53^ and Short Warwick-Edinburgh Mental Well-being Scale.^41^,^54^,^55^,^60^,^61^ Many studies had a specific level of cognition as an inclusion criterion, but only two studies^56^,^62^ reported a related instrument; however, the WEMWBS aims to “capture a wide conception of well-being, including … cognitive-evaluative dimensions.”^63^^,p.2^ Only one study reported on adverse events (unintended outcomes).^56^ Table 4 lists identified instruments within primary studies, alongside a description and some measurement properties. 

**Table 4:** List of instruments used in the primary studies with a description of the instrument and measurement properties, when available, for a general adult population

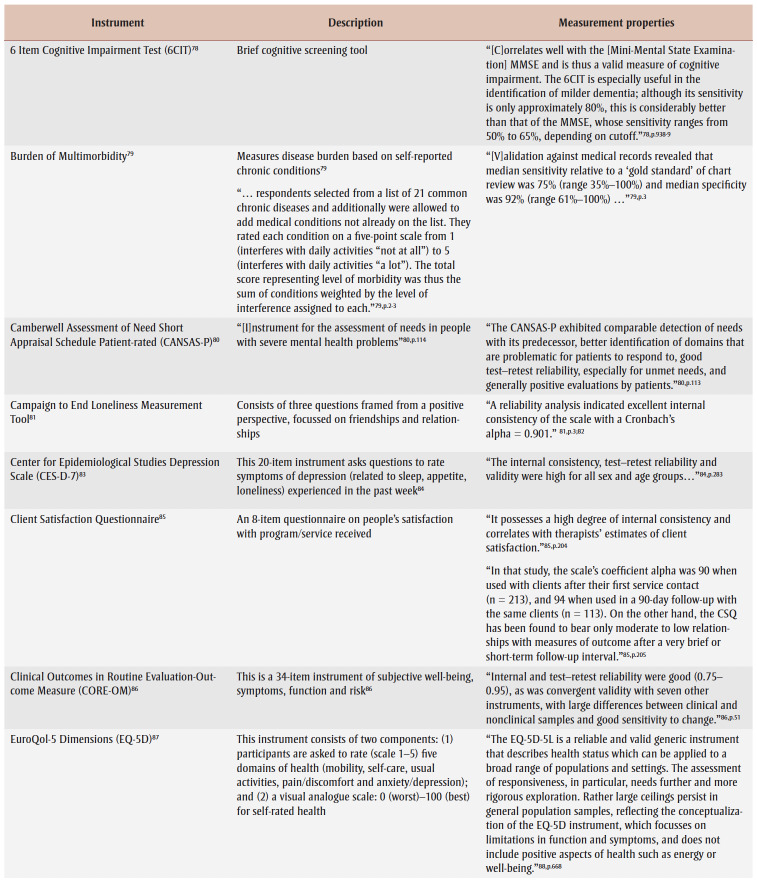 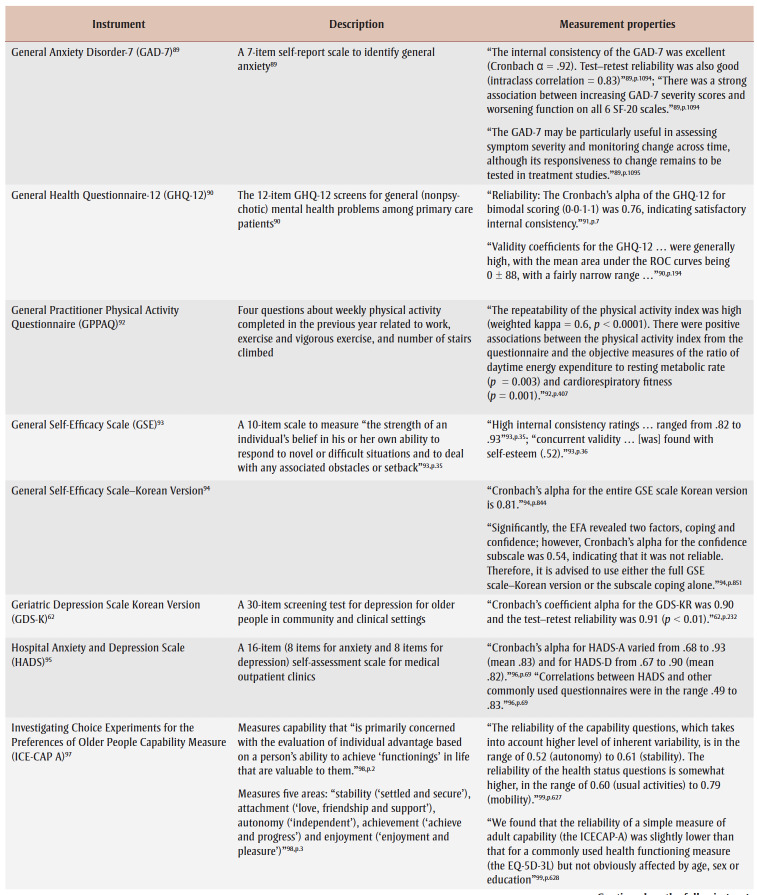 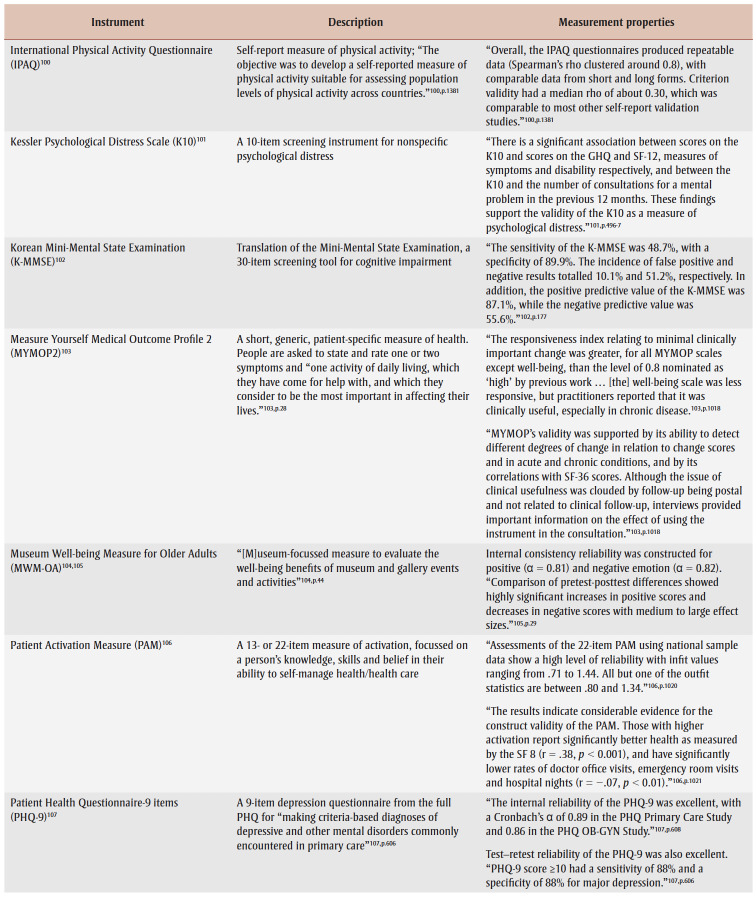 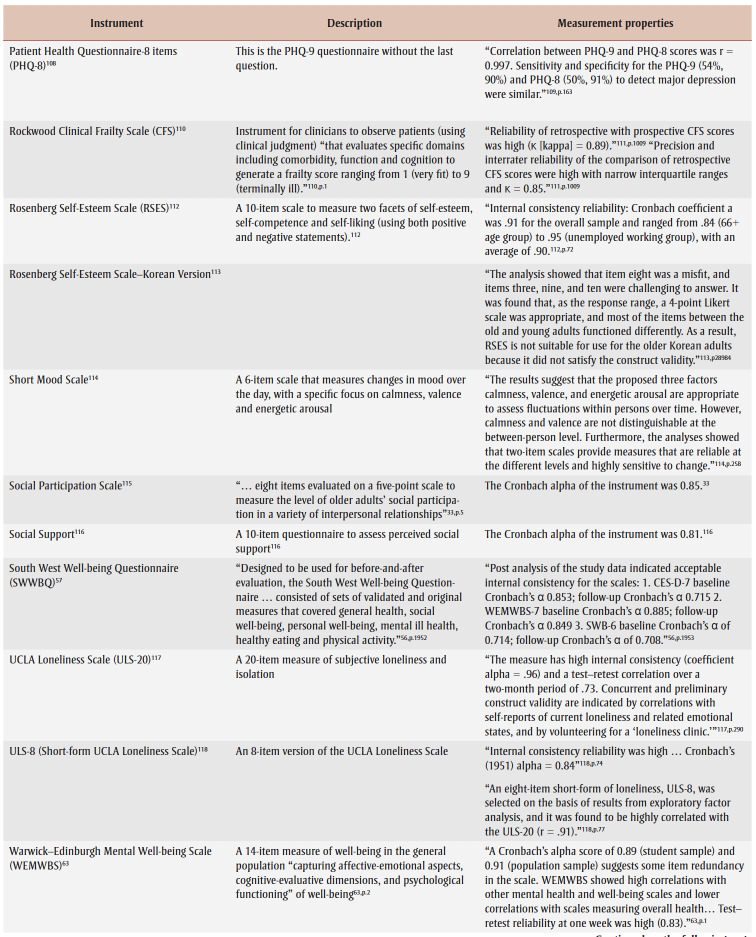 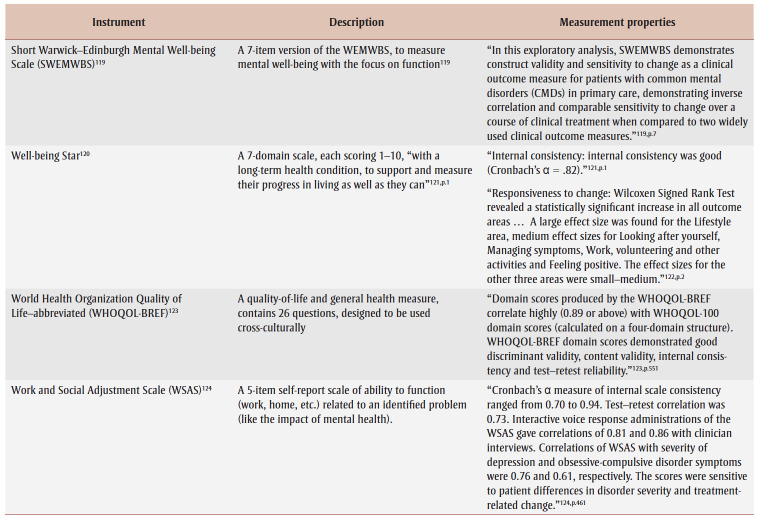

When comparing extracted outcomes (Table 3) with the NHS Social Prescribing Common Outcomes Framework,^14^ most categories were included by one or more primary studies. We observed that family caregivers, volunteers and voluntary groups were mentioned in many primary studies.^32^,^33^,^38^,^41^,^52^,^54^,^56^,^57^,^61^ Two primary studies^54^,^56^ included family caregivers within data collection, but we did not locate specific measures to evaluate volunteers’ experience or information related to societal or cost outcomes, despite the inclusion in the NHS framework. It is notable only six primary studies^46^,^50^,^52^,^53^,^57^,^58^ reported on physical activity (also mentioned in the NHS framework), and these studies were all published before 2020. However, the NHS Social Prescribing Common Outcomes Framework^14^ was dated June 2020 and pertains to the health and social care system in the UK. Therefore, not all included primary studies may have followed the framework (as a guideline) because of where the study was located (e.g. outside of the UK) and when it was published, as only 13 (39%) studies were published in 2020 or later. 

**Demographic information**

Reporting varied for information provided in primary studies (n=33). All primary studies but two^59^,^61^ reported participants’ age and gender/sex. Thirteen primary studies collected information on ethnicity/culture/language/race;^7^,^30^,^39^,^41^-^43^,^47^,^48^,^52^-^54^,^57^,^58^ 12 primary studies reported on occupation/employment;^30^,^35^-^37^,^39^,^42^,^46^,^48^,^50^,^52^,^58^,^60^ seven primary studies reported on socioeconomic status (based on place of residence);^34^-^37^,^50^,^53^,^54^ four primary studies reported on education;^33^,^40^,^52^,^58^ and four primary studies mentioned disability.^30^,^51^,^57^,^58^

## Discussion

We provide an overview of the outcomes and instruments used in peer-reviewed literature for social prescribing with adults aged 18 years and older, and highlight variability in the type and number of instruments used within studies. Our findings support and extend previous work^11^,^12^ by comparing identified outcomes with a published taxonomy.^13^ Many instruments were PROMs, alongside some PREMs, with less emphasis on physical activity, cognition or people providing unpaid care, such as family members and volunteers; only one study reported on unintended outcomes (e.g. harms). We further identified, similar to the recent mapping review,^12^ missing information related to equity: most studies only provided basic participant descriptive information, yet these data inform the development of equity within program development and delivery. Further, this evidence synthesis is a reminder for more comprehensive reporting of outcomes, given the recent development of reporting guidelines.^20^ Overall, this synthesis could be used to guide future research studies and program evaluations. It will be used to support our next phase—to conduct a modified Delphi study to determine outcomes important for people who impact or are impacted by social prescribing.^15^

***General interpretation***

Almost all taxonomy core areas and domains were included across social prescribing primary studies, but there was a strong emphasis on well-being and mental health. Noteworthy is the lack of studies measuring physical and daily activity to quantify any behaviour change associated with participation resulting from the prescription. However, it is possible, based on our inclusion criteria, that we excluded studies focussed on exercise and physical activity but that did not call their program “social prescribing.” There were included studies that focussed on other activities (e.g. arts, museum, connection) which could result in incidental physical activity. However, the studies did not routinely capture “what” people are doing within programs (e.g. incidental physical activity). Arts- or museum-based programs are not necessarily promoted as physical activity, but they are opportunities to add movement into everyday life.

Another identified gap was the measurement or collection of information on people’s cognition. Although more than 20 of the primary studies reported populations as middle-aged or older (40+ years), including nine studies of people aged over 60years, only two studies reported using an instrument to directly evaluate the effect or impact of social prescribing on cognition.^33^,^56^ There is an interplay between mental health (e.g. depression, anxiety), physical health and cognition. Depression in later life can increase the risk of dementia^64^ or frailty.^65^ Conversely, life satisfaction may prevent a decline in some measures of cognition.^66^ In 2019, there were approximately 57 million people (all ages) living with dementia globally;^67^ this number is projected to rise to 152.8 million people by 2050.^67^ Cognitive function and unmet social needs may be related, possibly due to the consequences of negative environmental factors, such as food insecurity or fewer neighbourhood resources.^68^ Further, a decline in cognition can challenge functional independence and completion of simple daily tasks. 

These factors, at a minimum, should be considered when co-creating an action plan to address unmet needs. In an ideal situation, it would be beneficial to see if social participation (as an aim of social prescribing) could mitigate the risks for cognitive decline. Some social prescribing activities, such as arts- and museum-based programs, may encourage concurrent cognitive and physical activities, which when combined in other research has been effective at promoting cognitive health.^69^ Taken together, better understanding a person’s cognition could assist when connecting them with a link worker, introducing community resources and services, and determining the effectiveness of the social prescription. 

The NHS Social Prescribing Common Outcomes Framework highlights collecting information on volunteers,^14^ but there were fewer evaluations of the impact or effect of family caregivers, volunteers and volunteer organizations,^41^,^54^,^56^ despite their being mentioned in many primary studies.^32^,^38^,^41^,^52^,^54^,^56^,^57^,^61^ Despite the important contribution of volunteers personally and economically,^70^ there are fewer published studies for volunteering and social prescribing; when they are available, they are focussed on volunteering as a “prescription.”^71^,^72^

There were also few mentions of family caregivers in primary studies,^54^,^56^ who play an essential role in providing supportive care. At a personal and societal level, the costs of family caregiving are high. There is the likelihood of caregivers experiencing high risk of physical and mental health challenges resulting in reduced quality of life.^73^ Further, in 2017, a report from the Canadian Imperial Bank of Commerce (CIBC) estimated caregiving costs Canadians CAD 33 billion annually for direct and indirect costs, such as out of pocket expenses (including paying for other providers) and time away from work; most affected are women and people with lower income.^74^ Given the benefits of volunteers and family caregivers at a personal and societal level, it is important to support, tailor, track and evaluate this important contribution to health and social models of care.

Social prescribing has a central theme of connection, for people with unmet social needs, family members, volunteers, providers and community organizations. The creation and sustainment of relationships between and across interested parties depends on effective communication and trust,^22^ among other factors. There are relational strategies and techniques to build intra- and interpersonal trust of people, providers and implementation teams,^75^ which can be used to generate effective changes in the adoption and sustainability of programs or clinical practices.^76^ We did not locate outcomes on relationships, but may have missed these data because the information is available in the unpublished literature or in studies using different methods. Future research could consider measurement of the development, strength and sustainability of relationships for people receiving social prescribing, and for people who deliver, manage and make decisions for its delivery across the continuum of care and sectors.

***Strengths and limitations***

This work has many strengths to contribute to the science and practice of social prescribing. Despite the comprehensive approach in this synthesis, we recognize several limitations. First, we made the decision to include only peer-reviewed studies that described their program as “social prescribing.” Social prescribing is a relatively new care model, but similar programs have existed for decades. However, using this criterion means we excluded studies that align with this model but do not call themselves social prescribing. Conversely, it could also mean we included studies that called their program social prescribing when it may not have been as closely aligned to the definition that is now published.^3^ In the recent social prescribing mapping review, the authors noted the challenges with screening studies to determine if the intervention was social prescribing.^12^ In our previous reviews^8^,^9^ we had a similar experience, and thus decided to only include studies described as social prescribing. 

Second, we only included peer-reviewed studies when searching for outcomes. We made this decision because many systematic reviews noted data were missing across studies (Table 1), and we wanted to compare reporting in the peer-reviewed primary studies, which may be more likely to follow research reporting guidelines. We acknowledge this means we may have missed other outcomes, especially as there are many social prescribing studies published in the grey literature. 

Third, we only included outcomes that were captured using quantitative strategies, and we may have missed information that was obtained via interviews and focus groups. Despite the important and rich data obtained through these methods, our findings may not have changed substantially, as studies in the current review included outcomes from almost all taxonomy domains. Nonetheless, concepts such as social connectedness may be better explored through qualitative methods, to better understand the effect of a complex intervention such as social prescribing. Our work highlights what is or could be measured—it does not limit how the outcome or domain should be measured. 

Fourth, our work is only descriptive and does not provide any information as to which outcomes should or should not be included in evaluating social prescribing interventions. This was intentional, because determining the scope and priorities of future evaluation should be a collaborative process based on needs, preferences and supporting information, which together with interested parties (such as people, families, providers and decision makers) can be used to advance the science and practice of social prescribing.

## Conclusion

We recognize it is impossible to measure everything in one study, but a core set of outcomes would benefit the field. Although the NHS has already provided outcomes to include in social prescribing evaluations,^14^ there remains the need to expand the list, standardize what and how we measure outcomes, and provide more information when describing people and processes for social prescribing. Specifically, consideration should be given to equity-considered guidelines such as PROGRESS-Plus^21^ to describe communities and people receiving and delivering social prescribing. It is also important to provide more information on unintended outcomes and the rationale and instrument measurement properties (reliability, responsiveness and validity, at a minimum).^77^ As there is now an international definition of social prescribing,^3^ it is important to use it to guide interventions and how they align (or do not). The current work is intended to prompt interest and action in the continued development of the science and practice underpinning social prescribing.

## Acknowledgements

Professor Ashe gratefully acknowledges the support of the Canada Research Chairs Program. Dr. Chudyk acknowledges the support from the Canadian Institutes of Health Research through the Patient-Oriented Research Awards – Transition to Leadership Stream, Phase 2 Award. Dr. Esfandiari acknowledges the postdoctoral fellowship provided by the Edwin S.H. Leong Centre for Healthy Aging.

***Funding***

We gratefully acknowledge funding for this project from The University of British Columbia Health Innovation Funding Investment Award and Social Sciences and Humanities Research Council.

## Conflicts of interest

The authors declare no conflicts of interest.

## Authors’ contributions and statement

MCA, EE—conceptualization.

MCA, IKS, HA, AC, EE—methodology, formal analysis.

MCA, IKS—writing—original draft.

MCA, IKS, HA, AC, EE—writing—review and editing.

MCA, IKS—visualization. 

All authors read and agreed to the published version of the manuscript.

The content and views expressed in this article are those of the authors and do not necessarily reflect those of the Government of Canada.
